# Preliminary Data Regarding the Alleviating Effects of Haloperidol and Risperidone on the Short-Term Memory and Associative Learning in a Zebrafish Model of Schizophrenia

**DOI:** 10.3390/ph18101548

**Published:** 2025-10-14

**Authors:** Petru Fabian Lungu, Luminita Diana Hritcu, Mircea-Nicusor Nicoara, Alexandra Savuca, Alexandrina-Stefania Curpan, Alexandru Ionut Chelaru, Corina Miruna Lungu, Bogdan Gurzu, Ioana-Miruna Balmus, Alin Ciobica, Gabriel-Ionut Plavan

**Affiliations:** 1 Department of Biology, Faculty of Biology, University “Alexandru Ioan Cuza” of Iasi, 700506 Iasi, Romania; lungufabian123@gmail.com (P.F.L.); mirmag@uaic.ro (M.-N.N.); alexandra.savuca@yahoo.com (A.S.); andracurpan@yahoo.com (A.-S.C.); alin.ciobica@uaic.ro (A.C.); gabriel.plavan@uaic.ro (G.-I.P.); 2 Department of Biological and Morphofunctional Sciences, College of Medicine and Biological Science, Stefan cel Mare University of Suceava, 720229 Suceava, Romania; 3 Department of Public Health, Faculty of Veterinary Medicine, University of Life Sciences “Ion Ionescu de la Brad”, Mihail Sadoveanu Street, No 3, 700490 Iasi, Romania; lumidih@yahoo.com; 4 Doctoral School of Geosciences, Faculty of Geography-Geology, University “Alexandru Ioan Cuza” of Iasi, 700506 Iasi, Romania; chelaru.alexandru@yahoo.com; 5 Department of Biology, Faculty of Sciences, University “Vasile Alecsandri” of Bacau, No 157, Marasesti Street, 600115 Bacau, Romania; 6 Department of Psychology, Faculty of Psychology and Educational Sciences, University “Alexandru Ioan Cuza” of Iasi, 700506 Iasi, Romania; mirunacorina.lungu@yahoo.com; 7 Department of Morfofunctional Sciences II, Faculty of Medicine, “Grigore T. Popa” University of Medicine and Pharmacy from Iasi, 16th Universitatii Street, 700115 Iasi, Romania; bgurzu@yahoo.com; 8 Department of Exact Sciences and Natural Sciences, Institute of Interdisciplinary Research, University “Alexandru Ioan Cuza” of Iasi, Carol I Avenue, 20A, 700505 Iași, Romania; 9 Center of Biomedical Research, Romanian Academy, Iasi Branch, Teodor Codrescu 2, 700481 Iasi, Romania; 10 Academy of Romanian Scientists, 3 Ilfov, 050044 Bucharest, Romania; 11 “Ioan Haulica” Institute, Apollonia University, Pacurari Street 11, 700511 Iasi, Romania

**Keywords:** schizophrenia, risperidone, haloperidol, ketamine, cognitive performance, learning and memory, anxiety, social behaviour

## Abstract

**Background:** Schizophrenia (SCZ) is a psychiatric disorder that negatively impacts patients’ quality of life, frequently inducing difficulties in managing day-to-day tasks. Current research is persistently working on finding therapeutic methods to alleviate the positive and negative symptoms, as well as the associated cognitive dysfunctions. Since the main therapeutic approach in SCZ is antipsychotics, the current study aimed to explore the effects of typical (haloperidol, HAL) vs. atypical (risperidone, RIS) antipsychotics on the cognitive functions in an animal model (*Danio rerio*) of SCZ, obtained by ketamine (KET) administration. **Methods:** The cognitive evaluation of the zebrafish was performed using memory and learning tests based on two stimuli: food and colours (i.e., T memory test and novel object recognition (NOR) test, respectively). **Results:** According to the behavioural analyses, HAL significantly enhanced the cognitive performances of the SCZ model, as compared to RIS. Nonetheless, HAL and RIS exhibited comparable effects on social behaviour in the SCZ model. Interestingly, both HAL and RIS enhanced the interest for the novel object in the NOR test in control individuals, but significantly decreased it in the SCZ model. The interaction between KET and RIS could exhibit sedative properties. **Conclusions:** Both typical (HAL) and atypical (RIS) antipsychotics alleviated cognitive, socio-affective, and decision-making impairments in a ketamine-based adult zebrafish model of schizophrenia. HAL was more effective, particularly in food-stimulated decision-making compared to novel object or social stimuli. Colour influenced behavioural responses, with silver linked to prey/feeding effects and red perceived as aversive. The KET–RIS combination induced exploratory impairments, possibly due to sedative effects. These findings highlight differential pharmacological and ethological modulation of schizophrenia-like behaviours.

## 1. Introduction

Being a psychiatric disorder that negatively impacts the patients’ quality of life, current research is persistently working on finding therapeutic methods to alleviate the positive and negative symptoms of schizophrenia (SCZ). However, recent reports have documented the occurrence of significant cognitive impairments associated with SCZ, scarcely targeted by pharmacological treatments [1,2,3]. Several reports have also suggested that antipsychotics could contribute to cognitive improvement, yet the mechanisms of action remain unclear [4,5]. Also, some differences regarding their efficiency were reported in typical versus atypical antipsychotics. In this way, a few studies have suggested that atypical antipsychotics could show better improvements in cognitive functions, as compared to atypical ones [6,7,8].

Haloperidol (HAL) is a first-generation butyrophenone antipsychotic widely used for its strong antagonism of dopamine D2 receptors [9], particularly in mesolimbic and mesocortical pathways [10]. By counteracting excessive dopaminergic activity, HAL is effective in treating psychosis, schizophrenia, and Tourette’s syndrome [11].

Risperidone (RIS) is an atypical antipsychotic of the benzisoxazole class, acting as a monoaminergic antagonist with high affinity for dopamine and serotonin receptors [12]. It also interacts with α_1_/α_2_-adrenergic and histamine receptors, while lacking activity at muscarinic sites [13]. At low doses, its primary action appears to be serotonin receptor blockade, though α_1_-adrenergic antagonism may contribute to cardiovascular effects such as hypotension [14,15].

Zebrafish (*Danio rerio*) are valuable animal models for neuropsychiatric and pharmacological research, as Grone and Baraban [16] and Gerlai [17] have demonstrated their capacity to execute complex behavioural tasks, despite some differences in neurotransmitter systems and brain architecture, as compared to mammalian brains [18]. Also, previous comparative studies have shown that zebrafish and rodent models exhibit notable behavioural similarities, such as memory, learning, emotional responses, and social interactions [19]. According to previous descriptions of SCZ animal models, learning and memory are critical behavioural components, as they reflect fundamental aspects of the disorder [20]. These impairments often manifest early in the disease, correlate with specific neurological changes, and present significant opportunities for therapeutic research. This facilitates the understanding of the fundamental causes of SCZ and the development of effective pharmacological approaches [20]. Additionally, administering pharmacological compounds to zebrafish is more straightforward and less invasive [21,22].

The relevant pharmacological effects of ketamine (KET) on zebrafish models’ behaviour, such as movement abnormalities mostly caused by excessive spinning and enhanced swimming capacity, were extensively described in KET zebrafish models [23,24]. Despite being currently used as an anaesthetic, KET has demonstrated the capacity to induce dissociative and psychedelic effects, such as hallucinations and cognitive impairments, symptoms that were noted in individuals with SCZ [25]. Previous animal model studies have demonstrated that KET could successfully induce some SCZ-like behavioural impairments [26]. As the disruptions in glutamate neurotransmission could play a crucial role in the pathophysiology of SCZ, the induction of SCZ-like symptoms with KET in different animal models may result from its antagonistic effect on the N-Methyl-D-Aspartate (NMDA) ion channel receptor, a subtype of the glutamate receptor system [23,27,28,29]. Furthermore, researchers attributed the strong correlation between the impairment of associative learning in SCZ patients and their ability to associate familiar objects with spatial places to disruptions in the glutamatergic system and subsequent impairments in hippocampal NMDA activity [30]. In this context, zebrafish could be trained to perform both classical and operant conditioning tasks [21], providing significant behavioural insights into common SCZ-like behavioural patterns. Furthermore, other negative symptoms of SCZ, including social withdrawal, which occurs in SCZ patients as a consequence of NMDA receptor antagonists, were successfully modelled by KET administration in zebrafish [23,31]. As socially mediated motor dysfunctions (hyperactivity in social contexts and hypoactivity in isolation) were noted in SCZ patients [31], zebrafish could be used to evaluate the behavioural responses to social stimuli based on well-developed social behaviour [32].

Thus, this study aimed to evaluate and describe the possible behavioural effects of HAL and RIS with regard to memory and learning in a KET-exposure-based adult zebrafish model of SCZ, as well as to compare the two mentioned antipsychotics on the cognitive effect, given that they are characterised as typical and atypical antipsychotics, respectively.

## 2. Results

### 2.1. T-Maze Memory Test

**Figure 1:**
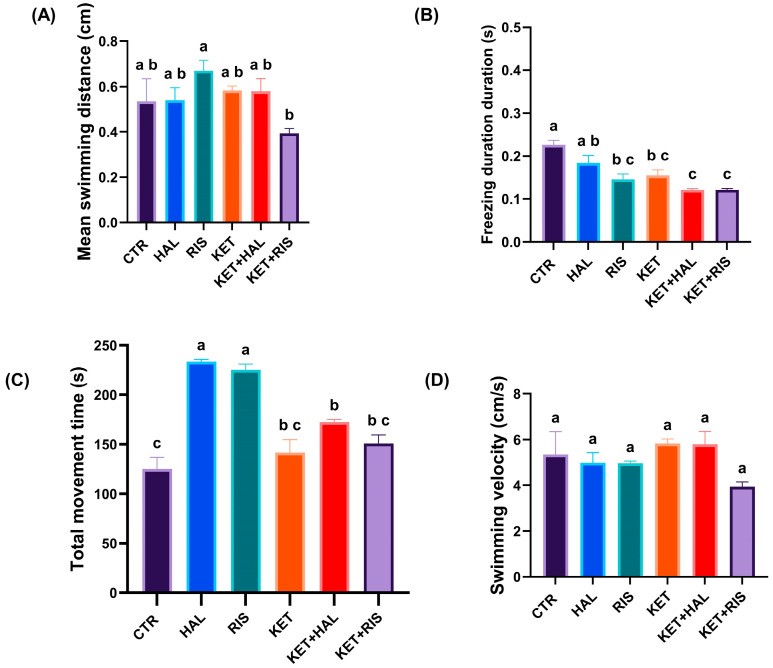
Behavioural assessment of memory and learning, as evaluated by the T-maze test (HAL = 0.13 mg/L; RIS = 0.08 mg/L; KET = 0.1 mg/mL). During a 5 min test trial, locomotion and anxiety-like behavioural parameters, such as (**A**) total swimming distance (cm), (**B**) freezing behaviour duration (s), (**C**) total movement time (s), and (**D**) swimming velocity (cm/s), were recorded and analysed. The results are presented as means ± SEM (*n* = 5 animals/group; a–c, Tukey’s HSD test).

The statistical analysis of our results showed that the mean swimming distance was significantly decreased for the KET + RIS group, as compared to the RIS group (*p* = 0.023) (Figure 1A). Freezing time was significantly lower in RIS (*p* < 0.001), KET (*p* = 0.002), KET + HAL (*p* < 0.001), and KET + RIS (*p* < 0.001), as compared to CTR (Figure 1B). The movement cumulative duration in the T-maze test was significantly higher in HAL (*p* < 0.001), RIS (*p* < 0.001), and KET + HAL (*p* = 0.008), as compared to CTR, while KET + HAL (*p* < 0.001) and KET + RIS (*p* < 0.001) showed significantly decreased activity, as compared to HAL and RIS groups, respectively (Figure 1C). No significant differences were found for the swimming velocity (Figure 1D).

**Figure 2:**
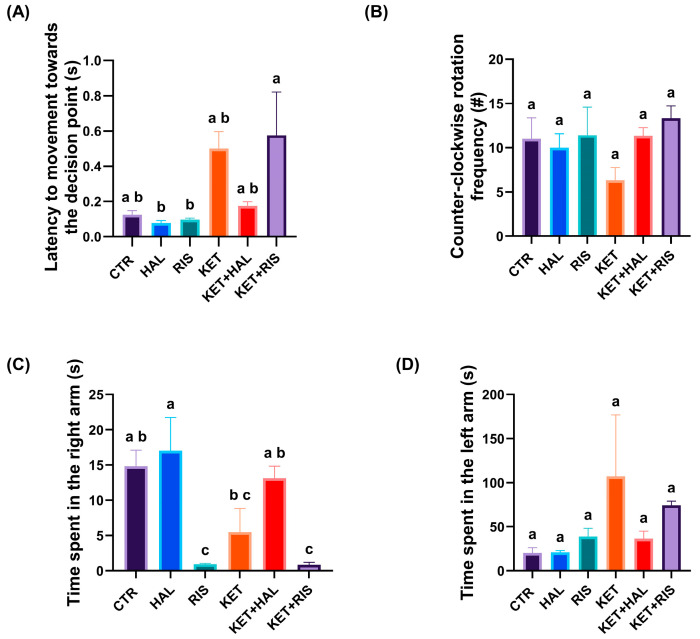
Behavioural assessment of memory and learning, as evaluated by the T-maze test (HAL = 0.13 mg/L; RIS = 0.08 mg/L; KET = 0.1 mg/mL). During a 5 min test trial, specific behavioural parameters, such as (**A**) latency to movement towards the decision point (s), (**B**) counter-clockwise rotation frequency (#), (**C**) time spent in the right arm (s), and (**D**) time spent in the left arm (s), were also recorded and analysed. The results are presented as means ± SEM (*n* = 5 animals/group; a–c, Tukey’s HSD test).

Regarding the specific behavioural parameters depicting the cognitive performance status, the movement latency towards the decision point was significantly higher in KET + RIS, as compared to the RIS group (*p* = 0.005) (Figure 2A). However, no significant differences were found between groups for counter-clockwise rotation frequency (Figure 2B). RIS (*p* = 0.012) and KET + RIS (*p* = 0.012) groups spent significantly less time in the right arm of the apparatus, as compared to CTR (Figure 2C), while the KET (*p* = 0.050) group spent significantly less time in the same arm, as compared to the HAL group. No significant differences were obtained while comparing the presence of the fish in the left arm (Figure 2D).

### 2.2. NOR Test

**Figure 3:**
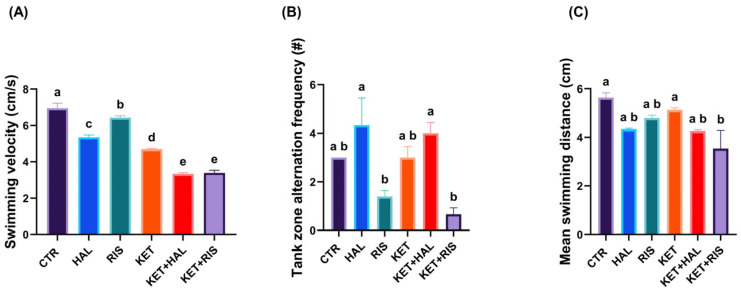
Behavioural assessment of memory and learning, as evaluated by NOR test (HAL = 0.13 mg/L; RIS = 0.08 mg/L; KET = 0.1 mg/mL). During the 5 min test trial, locomotion and anxiety-like behaviour parameters, such as (**A**) total swimming distance (cm), (**B**) swimming velocity (cm/s), and (**C**) tank zones alternation frequency (#), were recorded and analysed. The results are presented as means ± SEM (*n* = 5 animals/group; a–e, Tukey’s HSD test).

During the learning and memory task based on object recognition, we observed that HAL and RIS potentiated the effect of KET in decreasing the exploratory behaviour frequency (HAL vs. KET + HAL, *p* < 0.001; RIS vs. KET + RIS, *p* < 0.001; KET vs. KET + HAL, *p* < 0.001; KET vs. KET + RIS, *p* < 0.001) (Figure 3A). Also, RIS significantly inhibited locomotion in the KET-treated group (KET + RIS vs. KET, *p* = 0.02) compared to the CTR (*p* = 0.001) (Figure 3A). Swimming velocity was significantly lower after all the treatments except for RIS, as compared to CTR (*p_HAL_* < 0.001, *p_KET_* < 0.001, *p_KET + *HAL*_* < 0.001, *p_KET + RIS_* < 0.001). Also, lower swimming velocities were observed after HAL and RIS treatments in KET-treated groups (KET vs. KET + HAL, *p* < 0.001; KET vs. KET + RIS, *p* < 0.001) (Figure 3B). Zone alternation frequency, a relevant behavioural parameter for evaluating anxiety-like behaviour, was significantly decreased after HAL and RIS treatments in both healthy and KET-treated groups (HAL vs. RIS, *p* = 0.010; KET + HAL vs. KET + RIS, *p* = 0.003) (Figure 3C).

**Figure 4:**
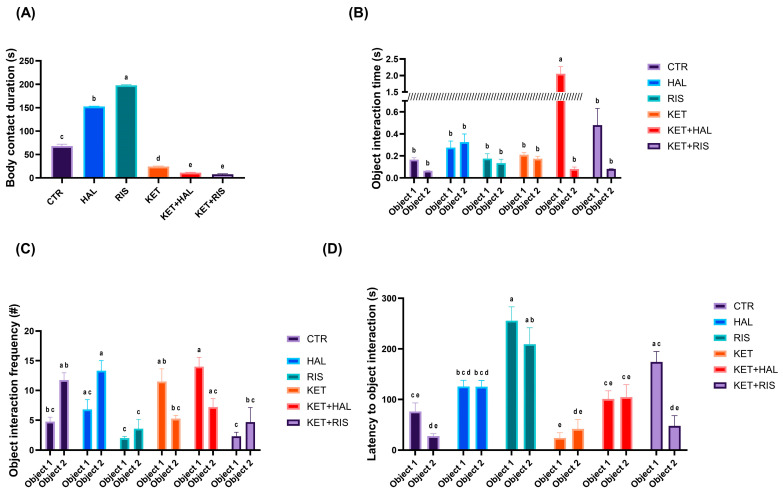
Behavioural assessment of memory and learning, as evaluated by the NOR test (HAL = 0.13 mg/L; RIS = 0.08 mg/L; KET = 0.1 mg/mL). During the 5 min test trial, specific behavioural parameters, such as (**A**) body contact duration (s), (**B**) object interaction time (s), (**C**) object interaction frequency (#), and (**D**) latency to the first interaction with objects (s), were recorded and analysed. The results are presented as means ± SEM (*n* = 5 animals/group; (**A**) a–e, Tukey’s HSD test; (**B**–**D**) a–e, Šídák’s multiple comparisons test).

Regarding the behavioural parameters describing the interaction with the objects, we observed that the time spent exhibiting interest for the spatial stimuli (body contact to the balls) was significantly increased after HAL and RIS treatments in healthy individuals (CTR vs. HAL, *p* < 0.001; CTR vs. RIS, *p* < 0.001), while the opposite effect was significantly noted in KET-treated groups (CTR vs. KET, *p* < 0.001; CTR vs. KET + HAL, *p* < 0.001; CTR vs. KET + RIS, *p* < 0.001) (Figure 4A). Furthermore, we observed that the interest in the silver ball was significantly higher in the KET + HAL group, as compared to the CTR (*p* < 0.001) and HAL (*p* < 0.001) groups. Also, we observed that RIS induced lower interest for the silver ball compared to HAL in KET-treated groups (KET + HAL vs. KET + RIS, *p* < 0.001) (Figure 4B). Silver object proximity was significantly higher after KET + RIS treatment, as compared to KET (*p* = 0.004) and KET + HAL treatments (*p* < 0.001). On the other hand, the interaction with the red ball was significantly less frequent in RIS and KET groups, as compared to CTR (*p_RIS_* = 0.019) and HAL (*p_RIS_* = 0.002, *p_KET_* = 0.021) (Figure 4C).

Meanwhile, the latency to interaction with the silver object was significantly higher in the RIS group, as compared to the CTR (*p_RIS_* < 0.001), HAL group (*p_RIS_* = 0.001), and KET group (*p_RIS_* < 0.001). Also, KET-treated animals exhibited more interest in the silver ball, as compared to HAL-treated animals (*p* = 0.039), while RIS treatment seemed to decrease latency to the interaction with the silver ball (*p* < 0.001). On the other hand, for the red ball (the new object), the latency to interaction was significantly higher after RIS treatment, as compared to CTR (*p* < 0.001), KET (*p* < 0.001), and KET + RIS (*p* < 0.001) (Figure 4D).

### 2.3. Social Interaction Test

**Figure 5:**
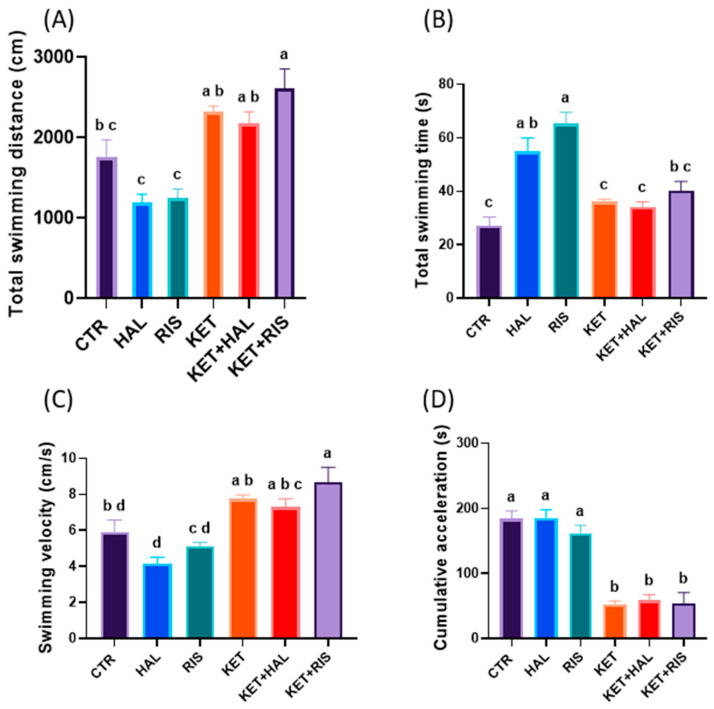
Behavioural assessment of sociability, as evaluated by the social interaction test (HAL = 0.13 mg/L; RIS = 0.08 mg/L; KET = 0.1 mg/mL). During the 5 min test trial, locomotion and anxiety-like behavioural parameters, such as (**A**) total swimming distance (cm), (**B**) total swimming time (s), (**C**) swimming velocity (cm/s), (**D**) cumulative acceleration (cm/s^2^), were recorded and analysed. The results are presented as means ± SEM (*n* = 5 animals/group; a–c, Tukey’s HSD test).

During the social interaction test, the mean swimming distance did not significantly differ after KET treatment, as compared to the untreated group. However, we observed that both HAL and RIS significantly decreased this parameter, as compared to untreated controls (*p_HAL_* < 0.001 *p_RIS_* < 0.001) (Figure 5A). Similarly, the total swimming time was not significantly influenced by KET treatment, but by HAL and RIS, as compared to untreated controls (*p_HAL_* < 0.001, *p_RIS_* < 0.001) (Figure 5B). When administered to KET-treated animals, RIS significantly increased the total swimming distance, as compared to untreated controls (CTR vs. KET + RIS, *p* = 0.011). Similar effects were noted for swimming velocity (CTR vs. KET + RIS, *p* = 0.009) (Figure 5C). However, we observed that the cumulative swimming acceleration was significantly decreased in all KET-treated groups, as compared to untreated controls (*p* < 0.001) (Figure 5D).

**Figure 6:**
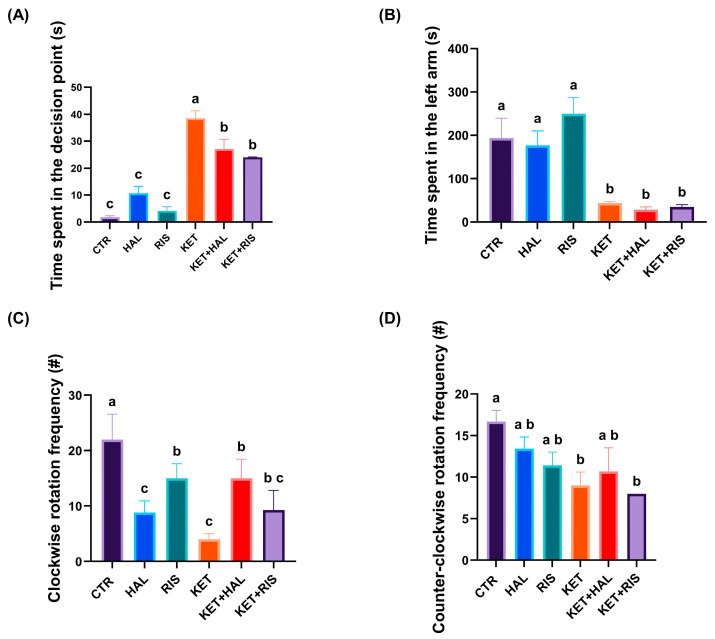
Behavioural assessment of sociability, as evaluated by social interaction test (HAL = 0.13 mg/L; RIS = 0.08 mg/L; KET = 0.1 mg/mL). During the 5 min test trial, specific behavioural parameters, such as (**A**) time spent in the decision point (s), (**B**) time spent in the left arm (s), (**C**) clockwise and (**D**) counter-clockwise rotation frequencies (#), were recorded and analysed. The results are presented as means ± SEM (*n* = 5 animals/group; a–c, Tukey’s HSD test).

In terms of specific parameters describing social interaction behaviours, we observed that the time spent in the decision point was significantly increased in all the KET-treated groups, as compared to the control group (CTR vs. KET, *p* < 0.001; CTR vs. KET + HAL, *p* < 0.001; CTR vs. KET + RIS, *p* < 0.001). Also, both HAL and RIS managed to decrease the decision time, as compared to the model group (KET vs. KET + HAL, *p* = 0.015; KET vs. KET + RIS, *p* = 0.001), but still remained significantly increased as compared to untreated controls (KET + HAL vs. CTR, *p* < 0.001; KET + RIS vs. CRT, *p* < 0.001) (Figure 6A). Similarly, the cumulative time spent in the left arm, the one containing the social stimuli, was significantly decreased in all KET-treated groups, as compared to untreated controls (CTR vs. KET, *p* = 0.009; CTR vs. KET + HAL, *p* = 0.004; CTR vs. KET + RIS, *p* = 0.005). HAL and RIS did not influence the preference for social stimuli, neither in KET-non-treated nor in KET-treated animals (Figure 6B). The frequency of clockwise rotations was decreased by KET treatment, as compared to untreated controls (CTR vs. KET, *p* < 0.001), suggesting a potential anxiety-like impairment. Also, HAL and RIS treatments both induced similar effects in healthy animals (CTR vs. HAL, *p* < 0.001; CTR vs. RIS, *p* = 0.018), but less pronounced than in the KET-treated group when referring to RIS treatment (RIS vs. KET, *p* < 0.001). HAL, but not RIS, significantly alleviated anxiety-like behaviour in KET-treated animals (HAL vs. KET + HAL, *p* = 0.045), yet not to CTR-comparable rates (CTR vs. KET + HAL, *p* = 0.018) (Figure 6C). The frequency of counter-clockwise rotations was significantly decreased by KET treatment and remained significantly low after RIS treatment, as compared to untreated controls (CTR vs. KET, *p* = 0.041; CTR vs. KET + RIS, *p* = 0.016) (Figure 6D).

## 3. Discussion

Memory and learning impairments often occur in SCZ patients, yet they are not the main objective of standard clinical pharmacotherapy. While antipsychotics are the most widely used therapeutic agents to alleviate positive and negative symptoms of SCZ, some studies in both patients and animal models of SCZ suggested that antipsychotics could be implicated in improving cognitive performances related to memory, learning, and spatial orientation [4,5]. The pharmacological mechanisms contributing to these effects are not fully understood and seem to depend on the pharmacological type (typical vs. atypical antipsychotics). For instance, previous studies have suggested that atypical antipsychotics exhibit superior efficacy in improving cognitive functions [7,22]. In this context, our study aimed to test this hypothesis and bring further evidence of the use of both typical and atypical antipsychotics in alleviating SCZ-associated cognitive impairments. We used a SCZ behavioural model based on exposing adult zebrafish to 0.1 mg/mL KET, as described before [26], to evaluate the effects of HAL and RIS, the most used typical and atypical antipsychotics, respectively, on the cognitive functions associated with some memory and learning processes often impaired in SCZ patients [12].

Our KET-based zebrafish model, previously behaviourally described and validated by our group [26], mirrors some of the trait behavioural features of SCZ, such as locomotor impairments suggestive of dissociative effects, confusion, and impaired decision-making cognitive processes. In this way, during all the behavioural assessment paradigms (T memory task, NOR task, and social preference task), we observed that the main locomotion parameters were significantly impaired in the KET-treated groups. Vinnakota et al. [33] explained that, in a mouse model, KET induced NMDA receptor changes to the GluN2D subunit (responsible for excitatory neurotransmission and synaptic plasticity), resulting in locomotion impairments and anxiety-related behaviour.

Moreover, the behavioural parameters associated with decision-making cognitive processes (such as latency to movement towards the decision point, time spent in the decision point, clockwise, and counter-clockwise rotations) were significantly and suggestively altered by KET exposure. In this way, behaviours such as excessive spinning and enhanced swimming capacity were also observed in our model, as also described by previous studies [23,24]. Another important feature that we successfully modelled by KET exposure could be correlated with associative learning impairments seen in SCZ patients. Due to glutamatergic impairment, both SCZ patients and KET-exposed zebrafish models exhibited slower spatial memory formation [34,35,36]. Our results showed that KET-exposed groups spent more time in the decision points as a result of impaired spatial memory regarding the position of the food stimuli, known or novel objects, and conspecifics. Chen et al. [37] previously explained that KET-addicted individuals with a history of psychotic episodes reported SCZ-like cognitive deficits, such as short-term memory and spatial problem-solving. While investigating the influence of colour on associative learning, Kim et al. [38] reported that colour-cued stimuli could enhance food reward-based associative learning. Our findings are consistent with previous research, which found that combined stimulation (food and colour) improved learning and could be useful in assessing memory status in cognitive decline-related zebrafish models [39,40].

Moreover, as often seen in SCZ patients, KET-exposed groups were characterised by social withdrawal, as described by the significantly decreased time spent in the left arm of the social preference task and frequency of the clockwise rotation behaviour, which is often considered as normal social behaviour for zebrafish larvae and adults [41,42]. Similarly, the significantly decreased duration of body contact behaviour suggests a decreased interest in external stimuli, disregarding their type (known or novel objects, or conspecifics).

We also observed beneficial behavioural effects following the KET treatment. Based on the sedative effects of KET, our results suggested that 0.1 mg/mL KET could mildly reduce anxiety-like behaviour, as seen during the behavioural assessment in locomotor parameters, such as increased total swimming distance. Zakhary et al. [23] and Riehl et al. [24] acknowledged this effect and assigned it to the cortisol-reducing properties of KET. Conversely, this same mechanism could also result in increased erratic movement behaviour in animal models, which often reflects the movement disorders reported in patients with schizophrenia (SCZ) [43]. Recent findings indicate that antipsychotic medications may also cause movement impairments, including extrapyramidal symptoms, with a greater prevalence associated with first-generation antipsychotics (such as HAL) due to their interaction with specific dopamine receptors. Prior research has indicated that the Taq1A A1 allele of the DRD2 gene and the 9-repeat allele of the DAT1 gene may constitute a risk factor for the emergence of movement impairment and extrapyramidal effects [44,45,46,47]. In animal models, while evaluating the behavioural responses to water deprivation in mice, Huang et al. [48] showed that compulsive behaviour was influenced by HAL in a dose-dependent manner. However, zebrafish response to HAL did not differ from that of rats in the water avoidance task, leading to the observation that the response is most likely attributable to GABAergic dysfunction rather than to movement, reward, and decision-making behaviour processes [36]. However, our results showed that the doses of HAL and RIS used in our study did not significantly induce erratic movements (as suggested by the frequency of counter-clockwise rotation behaviour or the cumulative acceleration), neither when administered to the SCZ model nor to healthy zebrafish. In addition, previous studies have shown that atypical antipsychotics are less predisposing to movement disorders and limiting anxious behaviour associated with SCZ positive symptoms [49,50,51]. In RIS-treated zebrafish, Idalencio et al. [52] have shown that RIS reduce the frequency of anxiety-like behaviour by cortisol level modulation, similar to KET. Our study also shows similar results, with both KET and antipsychotic-treated animals exhibiting less frequent anxiety-like behaviours, as compared to untreated controls. However, a significant reduction in anxiety-like behaviour (as described by relevant behavioural parameters, such as zone alternation frequency) was observed when KET–RIS-combined treatment was administered, suggesting an additive effect. Since the sedative effects of KET have already been described by previous research [24], together with our results in the current study, we observed that the combination of KET and RIS could be particularly beneficial in decreasing anxiety-like behaviour and minimising movement impairments. Our results are in agreement with Chen et al. [53], who previously documented the potential of RIS to inhibit hyperlocomotion in zebrafish. Arruda et al. [54] correlated the reduction in locomotor activity with the decrease in central nervous system excitability obtained by KET and RIS treatment. Further studies could, however, establish if tolerance would also develop in combined therapy, as seen in numerous antipsychotic agents, when the sedative effect decreases in a time-dependent manner [54]. Similarly, while evaluating the anxiolytic potential of antipsychotics, Hershenberg et al. [49] suggested that their efficacy could be limited by partial response and relapse risk, which can be particularly seen in RIS treatments.

Regarding the effects of antipsychotics on memory and learning processes, we observed that both HAL and RIS could improve cognitive performance in KET-treated animals. Seibt et al. [22] reported that zebrafish locomotor activity could be enhanced by pharmacological modulation, which could be a reflection of underlying cognitive changes. In our study, the use of a food-reward-based paradigm allowed the evaluation of associative learning and the influence of antipsychotics in an impaired SCZ-like animal model. For instance, our results suggested a complex drug interaction based on the increased time spent in the right arm (the food-stimulating arm) and highlighted an antagonistic effect of HAL relative to KET. Thus, this response may result from dopaminergic modulation, which could lead to distinct cognitive and behavioural outcomes, including the enhancement of associative memory, as explained by Arruda et al. [54]. Numerous prior studies have elucidated that the dysregulation of dopamine signalling, particularly via the D2 receptor antagonism by HAL, may rectify the anomalous reward-related processing induced by KET [55,56]. Conversely, prior research indicates that NMDA receptor hypofunction induced by KET may impair synaptic plasticity and memory encoding, thereby influencing the compensatory effects of antipsychotic treatment [57,58]. On the other hand, other studies [59] have reported that HAL treatment did not improve mice’s ability to retrieve information learned in memory tests. These controverted reports could be the result of a yet not fully understood mechanism of drug interaction [22].

Another important effect of the KET–RIS-combined treatment was obtained during the NOR test. In contrast to Gaspary et al.’s [60] original protocol, Magyary [61] has brought significant improvement by changing the static objects incorporated into the environment to floating objects on the water’s surface to stimulate feeding-associated behaviours with exploratory behaviours. Our results suggested that KET–RIS-combined exposure leads to markedly reduced exploratory behaviour, most likely due to RIS-induced freezing behaviours, as also observed in the T-maze memory task. Egan et al. [62] explained the occurrence of KET-induced freezing behaviour with KET’s anaesthetic properties, yet the current doses were chosen to avoid sedative effects. Rogóz [63] and Samizadeh et al. [64] did not associate reduced exploratory behaviour with antipsychotic treatments in the NOR task; thus, further studies should focus on elucidating controverted results. Previous studies demonstrate that the interaction between the NMDA receptor antagonism and serotonin–dopamine receptor blockade leads to a net reduction in exploratory behaviour, highlighting the intricate relationship between glutamatergic and dopaminergic systems in novelty recognition [65,66].

Another factor that could have been influencing the response of our animal models to the objects in NOR was the objects’ colours. Magyary [61] reported employing pink and the silver plastic spheres. Zebrafish tend to consume surface-dwelling insects that reflect light, thereby enhancing their interaction duration with the silver object [61,67]. Bollmann [68] also described this phenomenon, noting that varying object diameters can elicit hunting behaviour in zebrafish. On the other hand, our previous studies reported aversive behaviour of zebrafish model against red-coloured objects [26]. In addition, Michelotti et al. [69] described significant memory impairments and anxiety- and aggressiveness-like behaviours in KET-treated zebrafish models in relation to objects. Taken together with our results that showed that KET-treated animals exhibited increased interest in the silver object, this could indicate significant alterations in memory, learning, as well as in exploratory behaviour. However, we observed that the atypical antipsychotic (RIS) failed to alleviate these effects. On the contrary, we observed that the interest in the red sphere was significant, suggesting that RIS could potentiate the sedative effect of KET, as described before by our results and Pinheiro-da-Silva et al. [70]. In this context, our results suggested that the tested atypical antipsychotic could improve exploratory behaviour by decreasing anxious behaviour frequency, as compared to the evaluated typical antipsychotic, in a SCZ-like zebrafish model based on KET exposure.

Regarding the behavioural changes attributed to KET exposure, Riehl et al. acknowledged its potential to modulate locomotory performance in a social environment [24]. Moreover, our data showed that antipsychotic treatments led to significantly decreased swimming velocity, as compared to the control groups. Also, given that antipsychotics could exhibit similar effects, our results are in accordance with previous studies. In this way, several studies have shown that both HAL and RIS could decrease swimming velocity in both adult and larval zebrafish models [71].

Social interaction impairments are also very important components of SCZ-altered behaviour. In this way, both positive and negative symptoms of SCZ, as well as memory and learning impairments, could contribute to the occurrence of these alterations. Social detachment was previously described as typical behaviour for SCZ patients, yet little is known regarding the effects of antipsychotics in this regard. Our results suggested that KET-induced social interaction impairment could not be alleviated by the current doses of both the typical and atypical antipsychotics we tested. The marked avoidance of the social-stimuli-containing arm and the significantly increased presence of the KET-treated animals in the decision point during the social preference task, disregarding the antipsychotic treatment, could suggest that the social withdrawal could be due to memory and learning impairments. Several studies have previously described the correlation between social withdrawal observed in SCZ patients with impaired attention and altered verbal and working memory [72,73]. Furthermore, impaired social interaction was previously documented in zebrafish larvae treated with RIS [74], this being in accordance with some SCZ patient studies that have reported impaired social behaviour or no social benefits after antipsychotic treatments [75,76]. These alterations could reflect a convergence of NMDA receptor hypofunction, which disrupts cortical networks necessary for social cognition, with dopaminergic dysregulation, which may impair reward-related aspects of social interaction [57,77,78].

Regarding the changes in behavioural parameters for locomotion (total swimming distance, swimming velocity, and total mobility time), we observed that KET induced increased mobility in a social environment, as compared to food-based reward or novel objects. In this context, previous studies describing locomotor alterations in SCZ patients have suggested that hyperactivity in social contexts and hypoactivity in isolation could be typical manifestations [31]. Also, we observed that RIS, but not HAL, potentiated this effect, but not in a significant manner. Similar results were obtained by Celikyurt et al. [79] in a rat model of SCZ treated with RIS. This aspect could be a subject for further studies in evaluating the synergism between KET and RIS, as well as the social response of SCZ patients to atypical antipsychotics. SCZ patient studies have reported that atypical antipsychotics generally have fewer side effects on social status, yet do not bring significant improvements either [76,80,81,82]. By contrast, Terry Jr. et al. [83] showed that there are no substantial differences in social tasks and environmental recognition in a similar rodent model when receiving HAL and RIS at comparable doses. Thus, the mechanisms through which antipsychotics modify social interaction remain unclear and require additional research [84]. In relation to the changes occurring in the behavioural parameters depicting the nature of social interaction, Zaki et al. [85] observed that zebrafish larvae can interact with conspecifics in circular swimming patterns, ascribing this behaviour to typical interactions among zebrafish during clockwise rotations, behaviour that extends to maturity. During the social interaction task, we observed that the behavioural patterns associated with this social behaviour were significantly impaired following all the treatments, including KET and antipsychotics in healthy animals, suggesting a clear impairment in social interaction. Furthermore, neither HAL nor RIS managed to alleviate this side effect in KET-treated animals, despite the fact that the counter-clockwise rotation parameter was significantly decreased, depicting significantly decreased anxiety-like behaviour [41,42]. However, further studies are needed to explain these effects and interactions in zebrafish animal models.

Despite the promising results of our study, there are several limitations that are worth mentioning and discussing. Firstly, the small sample sizes are an important limitation that could have influenced the statistical power and interpretation of our results. However, given that the behavioural tasks required specific and different batches of animals to be tested to avoid supplemental stress and task avoidance, as well as unwanted tolerance to behavioural tasks (when novel stimuli were needed), and considering all the European and local ethical guidelines, we used the minimum accepted batch effectiveness in this preliminary study. Secondly, another limitation is the absence of chemical or genetic analyses that could further improve the discussion of the results and the formulation of hypotheses regarding the mechanisms of action for the receptors or neurotransmitters in question. These analyses require further validation of the described model, which is currently under development in our group. And last but not least, the administration method and the exposure duration of the pharmacological compounds through immersion could be an issue when considering the absorption by the organism; thus, additional analyses that could evaluate the amounts and targets of the drugs that are absorbed are further aims in developing the current model. Also, previous studies have suggested that the duration of exposure could influence the evaluated behavioural effects [39,40]. Additionally, the colour of the objects could contribute as a potential stressor that restricts proximal connection and inhibits exploratory behaviour. While zebrafish (*Danio rerio*) offer numerous advantages as a vertebrate model for neuroscience research, several important limitations warrant consideration. Structural and behavioural differences between zebrafish and mammals can influence the translational validity of findings. The zebrafish brain lacks certain higher-order cortical structures and complex laminar organisation characteristic of mammalian systems, which constrains its applicability in modelling advanced cognitive processes and executive functions [86]. Moreover, although zebrafish exhibit diverse behaviours related to learning, memory, anxiety, and social interaction, these behaviours may not fully replicate the complexity of mammalian social cognition or higher-level cognitive domains [87].

Nonetheless, zebrafish remain a valuable and emerging model for investigating the pharmacological effects of compounds relevant to neurodegenerative and neuropsychiatric disorders, including schizophrenia. Their small size, high fecundity, and permeability to water-soluble compounds enable high-throughput in vivo drug screening, allowing rapid and cost-effective evaluation of neuroactive agents and their behavioural consequences [87,88]. Despite these differences, zebrafish continue to bridge the gap between molecular and behavioural neuroscience, offering a tractable system for studying neural mechanisms and therapeutic targets when interpreted with due caution [86].

Our team intends to further examine the varying effects of typical and atypical antipsychotics on cognitive functions in schizophrenia as part of our ongoing research programme. Addressing the limitations identified in this study will enable us to produce more definitive and clinically significant insights regarding the impact of antipsychotic treatment on cognitive functions.

## 4. Materials and Methods

### 4.1. Animals

Eighty-five wild-type zebrafish adults were purchased from an authorised local breeder. The animals were acclimatised for 2 weeks prior to any procedures in 60 L glass tanks (water temperature 28 °C, 12:12 light cycle, water hardness 4–7 dGH, alkalinity 6–10 KH, pH 6–7.5, nitrites 0–10 NO_2_ mg/L, nitrates 0–2 NO_3_ mg/L, chlorine 0–0.2 mg/L). The animals were fed daily with a standard formula (TetraMin™ flakes, Herrenteich, Germany). Following the acclimatisation period, the fish were randomly assigned to experimental batches (10 L glass tanks, with similar conditions).

### 4.2. Ethical Note

The study was conducted according to the guidelines of the Declaration of Helsinki, and approved by the Ethics Committee of the Faculty of Biology, University “Alexandru Ioan Cuza” (no. 1851/15.05.2024), and the Ethics Committee of the Faculty of Veterinary Medicine, University of Life Sciences “Ion Ionescu de la Brad”, Iasi (no. 751/13.06.2023).

### 4.3. Substances and Treatments

KET solution was prepared extemporaneously using a commercial formulation (ketamine hydrochloride, 100 mg/mL, Pasteur Institute, Bucharest, Romania). The zebrafish were individually exposed to 0.1% KET in 50 mL flasks for five minutes. KET dose was selected based on prior experience and reduced to mitigate the anaesthetic effect [26]. HAL Sigma Aldrich, (Merck KGaA, Darmstadt, Germany)at a single dose of 0.13 mg/L or RIS Sigma Aldrich (Merck KGaA, Darmstadt, Germany) at a single dose of 0.08 mg/L [52,89] were administered similarly to the KET administration protocol. When exposed to both KET and antipsychotics, the animal models were first exposed to a 0.1% KET solution for 5 min, then to the designated antipsychotic. Antipsychotic doses were selected based on toxicity and intended not to induce developmental or hormonal changes [52,89]. The control group (CTR) was not exposed to any pharmacological compounds but was kept undisturbed in similar conditions to all the experimental batches.

Although direct pharmacokinetic validation is constrained, there exists corroborative evidence that waterborne delivery of antipsychotics and analogous compounds can produce consistent neurobehavioral and physiological effects in zebrafish. For example, immersion in risperidone modifies cortisol stress responses and locomotor activity [52,74], whereas haloperidol exposure in larvae results in significant developmental and behavioural alterations [90]. Likewise, ketamine delivered through immersion consistently produces antidepressant-like and locomotor effects, with recent research associating these results with alterations in CNS signalling [24]. The findings suggest that immersion is an effective method for generating neuroactive exposure in aquatic models.

### 4.4. Behavioural Tests

**Figure 7:**
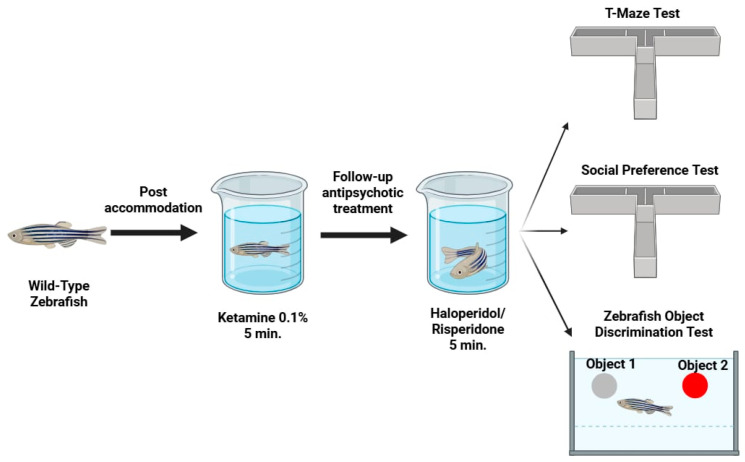
Experimental design representation.

Behavioural assessment was administered to evaluate features of memory and learning, affective behaviour, and social behavioural patterns (Figure 7).

#### 4.4.1. T-Maze Test

The T-maze test was designed for evaluating the cognitive performances of various animal models. In this study, the zebrafish were acclimatised in the T-maze apparatus for five days before being subjected to pharmacological interventions. The accommodation process included daily trials of descending duration and number of individuals, such as the following: days 1—zebrafish were left to freely explore the maze for an hour; day 2—groups of five fish were left to explore the maze for 30 min; days 3 and 4—groups of two fish underwent 5 min exploration trials; day 5—the fish were individually left to explore the maze for 5 min. The fish had access to the predetermined stimuli (food) each day in the right arm of the T-maze. Following the training session, the test trial was performed only once, on a specific group of fish designated for this test. One individual was released into the start arm and left to explore the maze freely for 5 min, with no food stimulation. During the test session, locomotor activity and exploratory behaviour (total swimming distance, cm; total movement time, s; swimming velocity, cm/s) as well as specific behavioural parameters for memory, learning, and anxiety (latency to movement towards the decision point, s; counter-clockwise rotation frequency; time spent in the right arm, s; time spent in the left arm, s; freezing behaviour duration, s) were recorded by an automated video tracking system and analysed with EthoVision XT 14.0 software (Noldus Information Technology, Wageningen, The Netherlands) [38].

#### 4.4.2. Novel Object Recognition (NOR) Test

**Figure 8:**
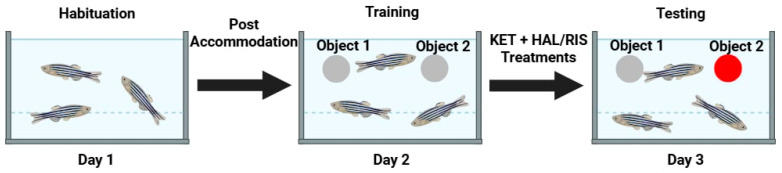
Graphical representation of the training phase in the novel object recognition (NOR) test.

The NOR test was performed according to the original Magyary protocol [61]. Following a 1-day habituation in the test tank (containing 6 L of water and no other objects), the exposure to two 10 millimetre silver balls was performed for 10 min. The next day, the silver spheres were inserted again and were also left in place for 10 min before being removed. On the third day, one of the silver balls was replaced by a red one, and the fish’s exploratory behaviour was recorded for 5 min (Figure 8). The behavioural parameters (total swimming distance, cm; swimming velocity, cm/s; tank zones alternation frequency; body contact duration, s; objects interaction time, s; object interaction frequency; latency to first interaction with objects, s) were analysed using EthoVision XT version 14.0 (Noldus Information Technology, Wageningen, The Netherlands) [61]. The NOR test was performed only once, on a specific group of fish designated for this test.

#### 4.4.3. The Social Preference Test

The social preference test was performed according to a protocol previously described by our group [39]. The test apparatus was a T-maze consisting of two equal-sided arms (left and right) and a longer start arm. The social stimulus was placed in the left arm of the T-maze, separated by a transparent PVC screen and consisted of a few conspecifics. Zebrafish were isolated by opaque white dividers placed between the tanks 5 days before the social interaction test. Only one such test was performed on the selected batches. EthoVision XT version 14 (Noldus Information Technology, Wageningen, The Netherlands) was used to record the exploratory behaviour for a 5 min test session [39] and behavioural parameters, such as total swimming distance (cm), total swimming time (s), swimming velocity (cm/s), cumulative acceleration (cm/s^2^), time spent in the decision point (s), time spent in the left arm (s), clockwise and counter-clockwise rotation frequencies, were recorded and analysed.

### 4.5. Data Analysis

The numerical data resulting from the behavioural assessment were statistically analysed using GraphPad PRISM software (version 10.0.0 for Windows, Boston, MA, USA). The results were expressed as means ± SEM. A one-way ANOVA test, followed by post hoc analysis (Tukey’s HSD test), were performed to test the differences between the groups. Additionally, a two-way ANOVA followed by Šídák’s multiple comparisons test was used to compare NOR behavioural parameters based on the interaction with the two objects used. A *p* ≤ 0.05 value was considered statistically significant.

## 5. Conclusions

In many cases, the memory and learning impairments co-occur in schizophrenia (SCZ) patients but remain unsolved by the standard clinical approach. Several reports have previously suggested that antipsychotics could also alleviate SCZ-associated cognitive impairments in a pharmacological and type-correlated manner (typical versus atypical). We found that both typical and atypical antipsychotics we tested (HAL and RIS, respectively) could alleviate cognitive impairments, socio-affective behaviour, and decision-making behaviour in a ketamine-based adult zebrafish model of SCZ. However, we observed that HAL, by contrast to RIS, was more effective, especially in alleviating food-stimulated decision-making behaviours, as compared to novel object or socially stimulated ones. Also, we found that colour is an important ethological factor contributing to stimulus response, such as depicting the correlation between the colour silver, prey effect, and feeding, by contrast to the colour red, which is often seen as aversive. Our results also suggested that the combination of KET and RIS produced exploratory impairments that could potentially be explained by sedative effects in zebrafish models.

## Data Availability

The data presented in this study will be made available on request from the corresponding author. The data are not publicly available due to [preliminary nature of the study and are part of an ample study].
